# Synchronous Quadruple Lung Cancer Treated Curatively by Photodynamic Therapy

**DOI:** 10.1155/DTE.3.115

**Published:** 1996

**Authors:** Makoto Saito, Harubumi Kato, Chimori Konaka, Tetsuya Okunaka, Kinnya Furukawa, Harumasa Sakai, Haruhiko Nakamura, Yoshihiro Ebihara

**Affiliations:** 1 Department of Surgery Tokyo Medical College 6-7-1 Nishishinjuku Shinjuku-ku Tokyo 160 Japan; 2 Department of Pathology Tokyo Medical College Tokyo 160 Japan

## Abstract

A 54-year-old male was diagnosed as having synchronous quadruple early stage lung cancer. All four tumors showed the same histologic type of in-situ or microinvasive squamous cell carcinoma, but existed independently in different bronchi. Photodynamic therapy of these four lesions was successfully performed by fiberoptic bronchoscopy because of the patient's poor pulmonary function. The patient is alive and well 51 months later.


*Diagnostic and Therapeutic Endoscopy*, 1996, Vol. 3, pp. 115–119
Reprints available directly from the publisher
Photocopying permitted by license only

© 1996 OPA (Overseas Publishers Association)
Amsterdam B.V. Published in The Netherlands
by Harwood Academic Publishers
Printed in Malaysia

### Synchronous Quadruple Lung Cancer Treated Curatively by Photodynamic Therapy

MAKOTO SAITO*, HARUBUMI KATO, CHIMORI KONAKA, TETSUYA OKUNAKA, KINNYA FURUKAWA, HARUMASA SAKAI, HARUHIKO NAKAMURA and YOSHIHIRO EBIHARA[a]

*Department of Surgery and [a]Pathology, Tokyo Medical College, Tokyo 160, Japan*

*(Received 1 May 1996; In final form 20 June 1996)*

**A 54-year-old male was diagnosed as having synchronous quadruple early stage lung cancer. All four tumors showed the same histologic type of in-situ or microinvasive squamous cell carcinoma, but existed independently in different bronchi. Photodynamic therapy of these four lesions was successfully performed by fiberoptic bronchoscopy because of the patient's poor pulmonary function. The patient is alive and well 51 months later.**

*Keywords:* Lung cancer, early stage, synchronous, quadruple, photodynamic therapy

### INTRODUCTION

Although no absolute criteria for the diagnosis of multiple lung cancer has been established, the vast majority of investigators employ Martini's criteria (Martini and Melamed, 1975; Verhagen *et al*, 1989). Most such lung cancers were double and a few were triple (Martini and Melamed, 1975; Verhagen *et al*, 1989; Deschamps *et al*; 1990). However, quadruple primary lung cancer has never been reported. A case of synchronous quadruple primary squamous cell carcinomas (SCC) of the lung treated nonsurgically with apparent curative effects by photodynamic therapy (PDT) is reported.

### CASE REPORT

A 54-year-old male had been followed up by annual check-up of sputum cytology from 1981 to 1989. The results had all been negative. However, in 1990, SCC was detected by sputum cytology and he was referred to our hospital for more detailed examinations. The patient had no complaints and no remarkable episodes. He had been smoking more than 20 cigarettes a day for 30 years (cigarette index more than 600). He was a road construction worker by occupation and therefore had been regularly exposed to dust for over 30 years. On admission, a chest roentgenogram demonstrated no abnormality, but emphysematous change in the

*Corresponding author.
Present address: Department of Surgery, Tokyo Medical College, 6-7-1 Nishishinjuku, Shinjuku-ku, Tokyo 160, Japan.

bilateral lung fields. A computed tomogram (CT) of the chest showed no extrabronchial mass. No distant metastasis was detected by CT of the brain and abdomen or by a bone scintigram. Fiberoptic bronchoscopy demonstrated no abnormal findings in the trachea and both main bronchi. The longitudinal mucosal folds were thickened at the orifice of the right upper lobe bronchus and the bronchial mucosa of the right B1, which was obstructed by extremely vicious mucus, was irregular (Fig 1a). After removing the mucus, the lesion was bronchoscopically identified in the orifice and surrounding mucosa of right B1, but did not extend peripherally beyond the segmental bronchus. Widened bifurcation and thickened bronchial mucosa was not detected in right B4 and B5 (Fig 1b). No abnormal findings were detected in the right lower bronchus. Enlargement of longitudinal folds at the orifice of the left upper division bronchus and a widened bifurcation between the left B1 + 2c and B3 were also recognized (Fig 1c). The left lingular lobe bronchus had no abnormal findings. The orifice of left B6 showed thickening of longitudinal folds and the bifurcation between the left B6a, B6b and B6c was swollen (Fig 1d). Left B8, B9 and B10 appeared normal bronchoscopically. Histological specimens from the tumors were obtained by bronchial biopsies from the bifurcation between right B1 and B2 (Fig 2a), between right B4 and B5 (Fig 2b), between left B1 + 2 and B3 (Fig 2c) and between left B6b and B6c. Fragments of bronchial mucosa obtained from all lesions except for the one from B6 included an in situ or microinvasive papillary proliferation of squamous epithelium, in which the structural arrangement was considerably disturbed and nuclear atypia of individual cells showed malignancy. Histological findings of the biopsy from the left B6 strongly suggested SCC, which was further supported by cytological specimens obtained from bronchial brushing of the lesion (Fig 2d). All four tumors were separately located at the orifices of the segmental bronchi while neither distant extension beyond segmental bronchi, nor proximal extension beyond lobar bronchi could be detected histologically. Therefore the patient was diagnosed as having synchronous quadruple SCC of the lung. Photodynamic therapy (PDT) was successfully performed in these four lesions through fiberoptic bronchoscopy because of the patient's poor pulmonary function which showed 3.6L in vital capacity and 108% in %VC, 1.2L in forced expiratory volume at 1.0 second and 35% in %FEV1.0 (Kato *et al*, 1990). 48 hours after the intravenous administration of 2.5 mg/kg of hematoporphyrin derivative (Photofrin II, Photomedica, Raritan, NJ) as photosensitizer, the lesions were illuminated by an eximer dye laser (PDT EDL-1, Hamamatsu Photonics Co., Shizuoka, Japan) with energy density of 125 Joules/cm2. Apparent complete remission of all four lesions was obtained, although the patient suffered from the complication of slight sunburn on exposed skin. The patient is alive 51 months after the treatment without any evidence of recurrence.

### DISCUSSION

Multiple primary lung cancer occurs either synchronously or metachronously in 1 to 3% of patients with lung cancer (Martini and Melamed, 1975; Verhagen *et al*, 1989; Deschamps *et al*; 1990). Synchronous lung cancer is less frequent than metachronous lung cancer (Martini and Melamed, 1975; Verhagen *et al*, 1989; Deschamps *et al*; 1990). Among synchronous lung cancers, the most frequent histologic type combination is double squamous cell carcinomas (Martini and Melamed, 1975; Verhagen *et al*, 1989; Deschamps *et al*; 1990). Synchronous cancer of similar histologic type can always be considered as possible metastasis within lungs or bronchi. However, many authors consider such tumors to be multiple primary cancers if they contain separate and independent areas of in situ carcinoma, if they occur in different lobes, or bilaterally, if they occur apart from regional lymphatics and if extrathoracic extent is negligible (Martini and Melamed, 1975; Deschamps *et al*; 1990). In this patient, SCC was cytologically detected after a nine-year period of annual check-up of sputum cytology. Since chest roentogenogram and CT of the chest were negative on admission, all bilateral visible bronchi up to subsegmental bronchi were examined by fiberoptic bronchoscopy. The findings

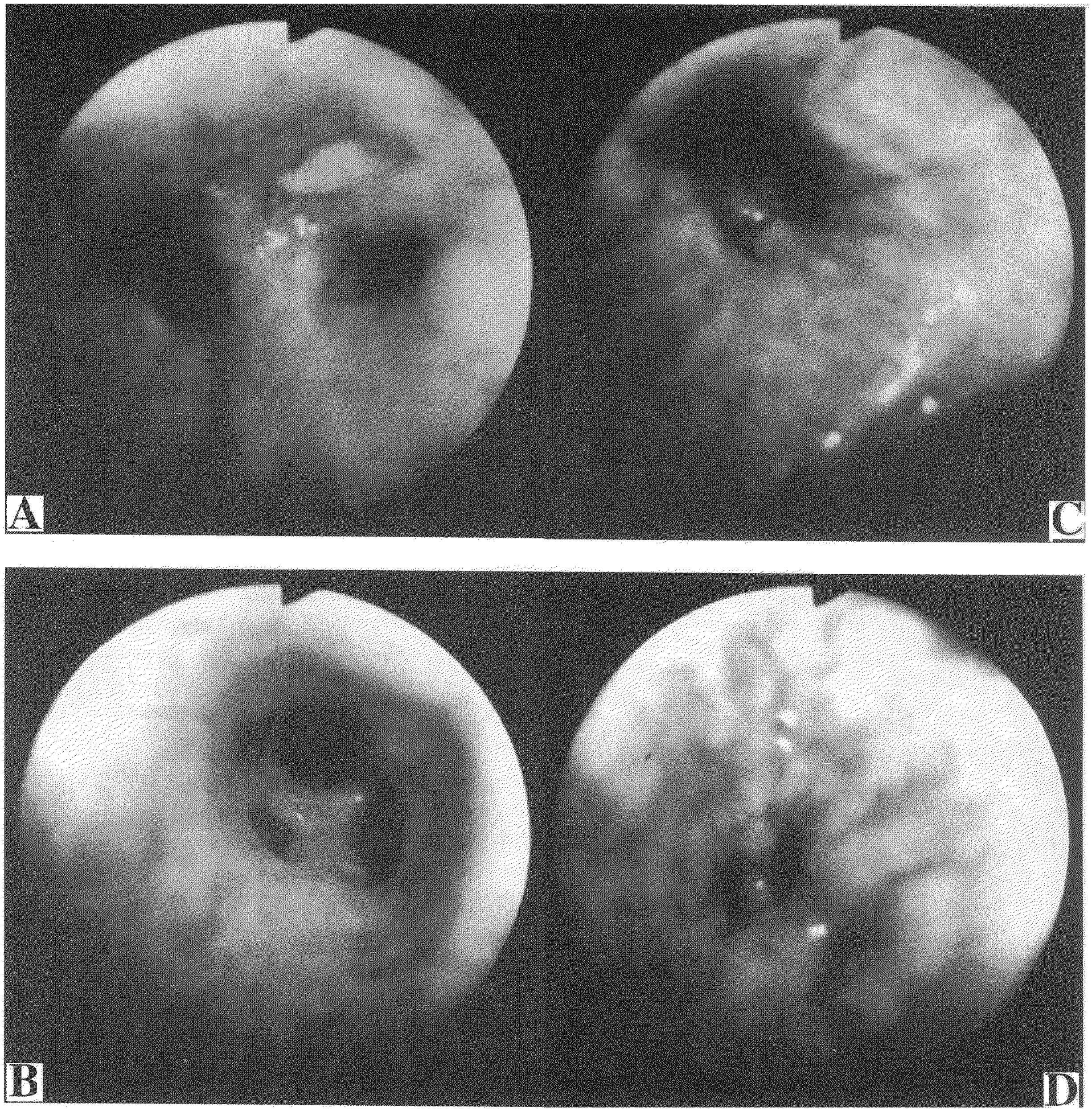

FIGURE 1 The bronchoscopic findings of the four lesions showed swelling of the bronchial mucosa in right B1 (a), the right middle lobe bronchus (b), the left upperdevision bronchus (c) and left B6 (d), with widened bifurcations of each segmental bronchi.

of the four lesions showed swelling of bronchial mucosa with widened bifurcation of segmental bronchi. All four tumors showed a similar histologic type of in-situ or microinvasive SCC, but were present independently in different bronchi. Therefore the patient was diagnosed as achieving synchronous quadruple SCC of the lung according to Martini's criteria (Martini and Melamed, 1975). At present, early stage lung cancer can be definitively diagnosed only by surgical specimens and/or autopsy

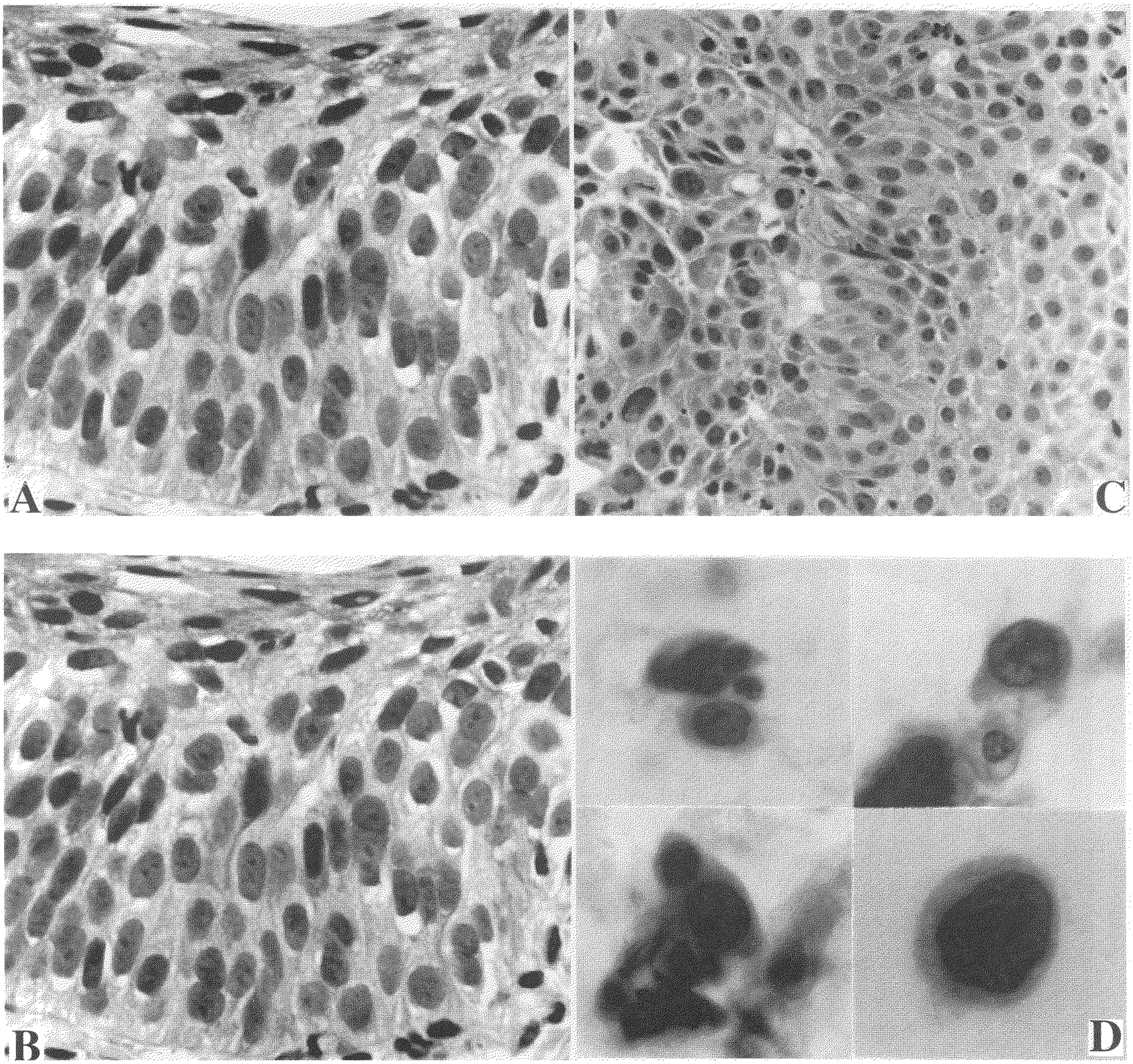

FIGURE 2 Histological specimens stained by haematoxylin-eosin from the bifurcations between right B1 and B2 (a, ×200), between right B4 and B5 (b, ×100), between left B1 + 2 and B3 (c, ×100), and cytological specimens stained by Papanicolaou's method from the bifurcation between left B6b and B6c (d, × 400).

examination. However, The Japan Lung Cancer Society proposed criteria for bronchoscopic evaluation of early stage lung cancer in 1987 (The Japan Lung Cancer Society, 1987).

According to those criteria, this patient can be considered to have had bronchoscopically defined early stage lung cancer. Sputum mass surveys for lung cancer are now being performed in several institutes and the early detection of central type early stage lung cancer, i.e. roentgenographically occult central type lung cancer, is becoming increasingly possible (Cortese *et al.* 1983). Patients with occult lung cancer require thorough examination of all bronchi by means of fiberoptic bronchoscopy, as multiple primary lung cancer may be present. Fiberoptic bronchoscopic findings of early stage central type lung cancer, most of which consist of squamous cell carcinoma, demonstrate only swelling of bronchial mucosa, with or without redness (Kato *et al*, 1990). All visible bronchi should be histologically/cytologically examined, to detect not only cancer foci, but also the extent of superficial invasion. Patients with multiple lung cancer may have been regularly exposed to carcinogens. Deschamps and his colleagues showed that 35 of 36 surgical patients with two synchronous primary lung cancer had smoked in the past, 27 of whom were still smoking at the time of surgery, and that nine of 36 patients later had a third primary lung cancer (Deschamps *et al*, 1990). Uyama *et al* diagnosed eight (8.9%) lung cancer patients in 90 workers exposed to chromate compounds (Uyama *et al*, 1989). Seven of eight patients smoked, of whom four patient had a cigarette index of more than 400. In three (37.5%) of eight patients, other lung cancer foci were detected during the postoperative follow-up by sputum cytology and bronchoscopy. Two of these three patients had multicentric cancer foci. Our patient had a cigarette index of more than 600 and was also exposed to dust which may have included carcinogens, i.e. tar or asbestos, for 30 years. The risk of developing other metachronous cancers still remains in this patient, therefore he requires aggressive follow-up. All cancer foci should be surgically resected if the pulmonary function is good. In the present patient, however, PDT was performed because of his poor pulmonary function. The authors have treated 21 inoperable patients with early stage lung cancer by PDT. Among 21 patients, nineteen patients are disease-free 10 to 82 months after treatment (Kato *et al*, 1990). In reviewing this case of synchronous quadruple SCC of the lung, all of which were at an early stage bronchoscopically. Combination of sputum cytology and fiberoptic bronchoscopy can be seen to be strongly important to detect central type early stage lung cancer, including multiple primary lung cancer and to follow up recurrence or development of other cancers.

### *References*

[1] Martini, N. and Melamed, M. R. (1975). Multiple primary lung cancers. *J. Thorac. Cardiovasc. Surg.*, **70**, 378–385.

[2] Verhagen, A. F., Wal, H. J. V. D., Cox, A. L. and Lacquet, L. K. (1989). Surgical treatment of multiple primary lung cancers. *Thorac. Cardiovasc. Surgeon*, **37**, 107–111.

[3] Deschamps, C., Pairolelo, P. C., Trastek, V. F. and Payne, W. S. (1990). Multiple primary lung cancers: results of surgical treatment. *J. Thorac. Cardiovasc. Surg.*, **99**, 769–78.

[4] Kato, H., Konaka, C., Kinoshita, K. *et al.* (1990). Laser endoscopy with photodynamic therapy in the respiretory tract. In: Oguro N. and Takagi K., eds. *Endoscopic approaches to cancer diagnosis and treatment.* Tokyo: Scientific Societies Press, 139–152.

[5] The Japan Lung Cancer Society. (1987). General rule for clinical and pathological record of lung cancer (in Japanese). Tokyo: Kanehara Shuppan, 64–68.

[6] Cortese, D. A., Pairolero, P. C., Bergstralh, E. J. *et al.* (1983). Roentogenographically occult lung cancer: a ten-year experience. *J. Thorac. Cardiovasc. Surg.*, **86**, 373–380.

[7] Uyama, T., Monden, Y., Tsuyaguchi, M. *et al* (1989). Lung cancer in chroma te workers: high-risk group for multiple lung cancer. *J. Surg. Oncol.*, **41**, 213–218.

